# Protocol for a clinically annotated biorepository of samples from Australian immune-compromised patients to investigate the host–microbiome interaction

**DOI:** 10.1136/bmjopen-2024-085504

**Published:** 2024-09-12

**Authors:** Olivia C Smibert, Jason A Trubiano, Jason C Kwong, Kate A Markey, Monica A Slavin

**Affiliations:** 1 Peter MacCallum Cancer Centre, Melbourne, Victoria, Australia; 2 Sir Peter MacCallum Department of Oncology, University of Melbourne, Melbourne, Victoria, Australia; 3 Department of Infectious Diseases, Peter MacCallum Cancer Centre, Melbourne, Victoria, Australia; 4 Department of Infectious Diseases & Immunology, Austin Health, Melbourne, Victoria, Australia; 5 National Centre for Infections in Cancer, Peter MacCallum Cancer Centre, Melbourne, Victoria, Australia; 6 Department of Infectious Diseases, University of Melbourne, The Peter Doherty Institute for Infection and Immunity, Melbourne, Victoria, Australia; 7 Centre for Antibiotic Allergy and Research, Department of Infectious Diseases, Austin Health, Heidelberg, Victoria, Australia; 8 Department of Microbiology & Immunology, The Peter Doherty Institute for Infection and Immunity, The University of Melbourne, Melbourne, Victoria, Australia; 9 Translational Science and Therapeutics Division, Fred Hutchinson Cancer Center (FHCC), Seattle, Washington, USA; 10 Department of Medicine, University of Washington, Seattle, Washington, USA

**Keywords:** TRANSPLANT MEDICINE, BACTERIOLOGY, IMMUNOLOGY, INFECTIOUS DISEASES, Transplant medicine

## Abstract

**Introduction:**

The human gut microbiota has the potential to modulate the outcomes of several human diseases. This effect is likely to be mediated through interaction with the host immune system. This protocol details the establishment of a biorepository of clinically annotated samples, which we will use to explore correlations between the gut microbiota and the immune system of immune-compromised patients. We aim to identify microbiome-related risk factors for adverse outcomes.

**Methods and analyses:**

This is a protocol for the development of a biorepository of clinically annotated samples collected prospectively across three centres in Melbourne, Australia. Participants will be recruited across the following clinical streams: (1) acute leukaemia and allogeneic stem cell transplant; (2) end-stage liver disease and liver transplant; (3) patients receiving any cancer immunotherapies (eg, chimeric antigen receptor therapy); (4) deceased organ donors and (5) healthy adult controls. Participants will be asked to provide paired peripheral blood and microbiota samples (stool and saliva) at either (1) single time point for healthy controls and deceased organ donors or (2) longitudinally over multiple prespecified or event-driven time points for the remaining cohorts. Sampling of fluid from bronchoalveolar lavage and colonoscopy or biopsy of tissues undertaken during routine care will also be performed.

**Ethics and dissemination:**

Ethical approval has been obtained from the relevant local ethics committee (The Royal Melbourne Hospital Human Research Ethics Committee). The results of this study will be disseminated by various scientific platforms including social media, international presentations and publication in peer-reviewed journals.

**Trial registration number:**

ACTRN12623001105639. Date registered 20 October 2023.

**STRENGTHS AND LIMITATIONS OF THIS STUDY**

- Sampling of the gut microbiome and host immune response before, during and after transplant or immune therapy will allow detailed phenotyping to better characterise the clinically relevant microbe–microbe and host–microbe interactions.
- The same types of samples will be collected from clinically distinct cohorts which will allow a contrast of microbiota and host interactions at clinical time points common to all (ie, infection or immune-related event).
- This protocol represents the first description of the deceased organ donor enteric microbiota samples that will link with the respective recipient’s enteric microbiota samples.
- Comprehensive dietary history, via a novel technology, prior to sample collection represents an important opportunity to investigate the extent to which dietary factors confound observations of the microbiota.
- The conclusions from this study may not be generalisable to other immunocompromised cohorts due to the influence of geographical, dietary and local treatment protocols which differ between populations.

Despite advances in anti-infective strategies, there remains a significant burden of infection from a broad range of pathogens in patients with cancer and organ transplant recipients. New approaches to prevention and treatment are urgently required. Infection is the leading cause of mortality at day 100 after allogeneic stem cell transplant.^1^ Infection is also the single greatest cause of morbidity and mortality after liver transplantation with infection occurring in up to 50% of recipients and the cause of death in up to 9% of instances.^2^ Immune-based cancer therapies, including chimeric antigen receptor therapy (CAR-T), are transforming the management of B cell leukaemias and lymphomas and their use is being extended to other malignancies.^3 4^ However, infections occur commonly in 20%–40% of CAR-T cell recipients by 30 days after infusion.^5 6^

The gut microbiome has shown the potential to modulate outcomes of several human conditions including acute leukaemia and allogeneic stem cell transplant, a variety of cancers and after solid organ transplant.^7^,^11^ This effect is likely to be mediated through interaction with the host immune system and the intestinal microbiome is a promising target for biomarker discovery and therapeutic manipulation.^10 11^ Specific bacterial microbiome signatures have been associated with risk of infections and immunological complications after transplant and immune therapies in previous studies.^7 8 12 13^ Loss of diversity and expansion of pathobionts in faecal samples from patients undergoing induction chemotherapy for acute myeloid leukemia (AML) and allogeneic stem cell transplant correlate with an increased risk of subsequent microbiologically diagnosed infection.^814^,^16^ Risk of acute cellular rejection and bloodstream infection after liver transplant has been associated with loss of microbiome diversity and expansion of pathobionts.^17^,^19^ While prior investigations of the microbiome in transplant and cancer have identified correlations between microbial features and clinical outcomes, more detailed host immune profiling and frequent stool sampling is required to draw more meaningful conclusions about the mechanisms underlying the host–host and host–microbe interaction. The Host Microbiome in Specialty Patient Populations (HOMISPEC) protocol aims to address some of these limitations with frequent longitudinal and event-driven sampling in addition to sampling from multiple compartments (blood, saliva and tissues) that will allow detailed interrogation of these host–microbe interaction and the between microbe interactions.

Given the association between the microbiome and disease in a broad range of pathologies, strategies for microbiome ‘optimisation’ are likely to improve patient outcomes. These may include individualised prophylaxis, precision antibiotic stewardship and microbiome-targeted interventions including dietary modification, prebiotics and faecal microbial transplant. Definition of the mechanisms through which microbes interact with each other and the host is essential before microbiome-directed therapies can be translated into clinical care.^20^ The objective of the HOMISPEC study is to establish a biorepository of clinically annotated samples from which a detailed exploration of the gut microbiota as both a biomarker of subsequent infection and a potential target for therapeutic manipulation can be explored.

## Methods and analyses

### Study design and setting

This is a protocol for the development of a biorepository of clinically annotated samples collected prospectively across three centres in Melbourne, Australia. The protocol will consent participants across the following three sites: Peter MacCallum Cancer Centre (Melbourne, Victoria, Australia), Austin Health (Melbourne, Victoria, Australia) and Royal Melbourne Hospital (Melbourne, Victoria, Australia) and in collaboration with the Australian Donation and Transplantation Biobank (ADTB). The Peter MacCallum Cancer Centre is one of only two public cancer specialty hospitals in Australia while Austin Health is a tertiary referral centre for liver and haematopoietic stem cell transplantation. The ADTB is an investigator-led biobank undertaken in collaboration with the Victorian organ procurement agency (DonateLife Victoria) and the only donor tissue biobank in Australia.

#### Outcomes

The objective of the HOMISPEC study is to establish a biorepository of clinically annotated samples to explore the interaction between the host and the microbiota in the setting of organ transplant and immune compromise.

The outcomes of interest to be explored across patient cohorts will be clinically or microbiologically defined infection and clinically or histopathologically defined immune events. The occurrence, timing and outcomes of infectious and immunological complications will be determined, defined and graded according to the following internationally accepted criteria: (1) European Organisation for Research and Treatment of Cancer/Invasive Fungal Infections Cooperative Group criteria for definition of invasive fungal disease^21^; (2) study^22^; (3) International Immunocompromised Host Society classification of microbiologically defined, clinically defined infections and fever of unknown focus^23^; (4) grading of severity from Common Terminology Criteria for Adverse Events^24^; (5) the Mount Sinai Acute GVHD International Consortium (MAGIC) consortium acute graft versus host disease (GVHD) criteria^25^; (6) Banff schema for grading liver allograft rejection^26^; the American Society for Transplantation and Cellular Therapy (ASTCT) criteria for Cytokine Release Syndrome (CRS) and immune effector cell-associated neurotoxicity syndrome (ICANS).^27^

### Participants and eligibility criteria

We will include participants across the following disease cohorts: (1) acute leukaemia and allogeneic stem cell transplant (HSCT); (2) end-stage liver disease and waitlisted for liver transplant; (3) recipients of cancer immune therapies (eg, CAR-T therapy); and (4) healthy adult controls. Controls will be identified from screening as those without known medical comorbidities and those without either overseas travel or antibiotic exposures during the preceding 12 months. Participants will be excluded if (1) age <18 years; (2) either they or their medical treatment decision-maker are unable or unwilling to provide written and informed consent. Potentially eligible patients will be identified from transplant and cancer service coordinators or outpatient clinics and will be approached and invited to participate. Deceased organ donor samples will be procured through collaboration with the Australian Donation and Transplant Biobank (ADTB). Participants will be followed up to 24 months post enrolment or withdrawal of participation, retransplant with the same organ which will be classified as graft failure and the end of follow-up or death. Patients who undergo retransplant for graft failure and are not already enrolled in the study will be eligible to participate. Patient follow-up visits will coincide with routine clinical visits which will limit loss to follow-up, particularly for participants who live in remote or regional parts of Australia. Healthy controls and deceased organ donors will have clinical data collected at a single time point only and will not undergo any follow-up.

### Patient and public involvement

Patients play a role in the organisation of the study and the development of the instructional material that is provided to participants to assist with home sample collection. A broad range of disciplines including specialty medical teams, nursing staff, dieticians, pharmacists, laboratory scientists, medical student and clinical coordinators have assisted with the organisation and implementation of the study.

### Data sources and measurements

#### Biorepository

**Figure 1:**
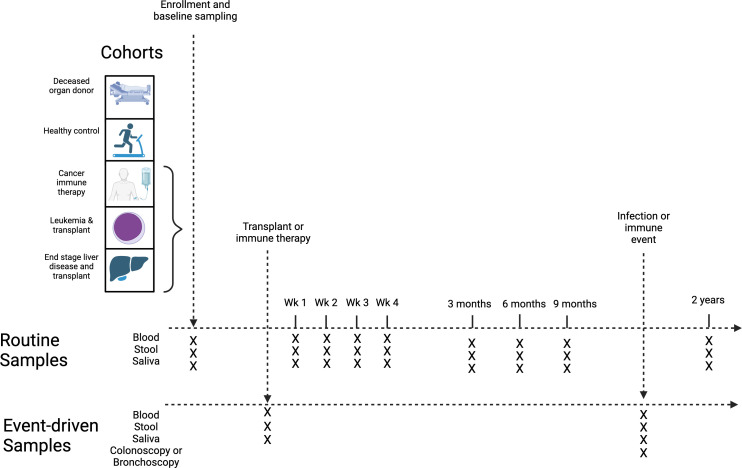
Summary of HOMISPEC study design.

Participants will be invited to provide stool, saliva and peripheral blood samples at enrolment and prespecified routine time points for up to 2 years (figure 1, online supplemental table 1). Two 9 mL blood samples will be collected from participants at each visit and conveyed immediately to the laboratory on ice (table 1). Tubes will be centrifuged at 4°C at 1500g from 12 min and plasma will be aliquoted and stored at −80°C while peripheral blood mononuclear cells (PBMCs) will be stored in liquid nitrogen. Participants will be instructed to collect a faecal and saliva sample at each time point. Written and illustrative instructions will be provided at each time point with a ‘stool collection kit’ and a ‘saliva collection kit’. Each stool kit will include an easy-to-use faecal collection method (DNAgenotek OM-AC1 Toilet Accessory), faecal collection tubes and instructions regarding how to collect the sample (table 1). Each saliva kit will include a saliva collection device and instructions regarding how to collect the sample (table 1). The samples may either be collected in the hospital or as an outpatient. If collected during a hospital visit, the sample will be conveyed immediately to the laboratory for batch processing. If the sample is collected at home, then it will either be transported to an upcoming scheduled visit for the study or returned via express post directly to the laboratory. Samples will be stored at room temperature and aliquoted and stored at −80°C within 7 days of collection. Additional samples of paired blood and stool/saliva will be collected at times of clinically relevant infection or immunological complications that will be notified to the investigators via the primary clinical teams. During routine clinical care, additional samples of fluid or tissue may also be collected from bronchoalveolar lavage, colonoscopy or other invasive procedures (table 1). Tissues from deceased organ donors providing liver allografts for participants recruited in cohort 2 will be collected through research agreement with the ADTB. Fresh tissue will be procured at the time of organ procurement by the transplant surgical team and conveyed with the donor organ per institutional policy of static cold storage to the primary study site for immediate processing and storage. Where possible, blood samples will be drawn contemporaneously with routine clinical blood tests. Biological samples will be processed and stored at the site of recruitment and will be batch analysed.

10.1136/bmjopen-2024-085504 online supplemental table 1

**Table 1:** Summary of HOMISPEC laboratory Procedures

Study subject	Research sample type	Time points	Sample quantity	Sample transport to biorepository	Sample collection	Processed sample	Sample aliquots	Storage temperature
Disease cohort (liver disease and haematological malignancy)	Peripheral blood	Baseline, week 1, 2, 3, 4, month 3, 6, 9, 12, 24	18 mL	2–8°C, ice packs	9 mL EDTA	Plasma	6 × 1.5 mL aliquots	−80°C
						PBMC	5 × 5 × 106/1 mL aliquots	Liquid nitrogen
	Stool	Baseline, week 1, 2, 3, 4, month 3, 6, 9, 12, 24	25 g	Ambient	Sterile sample contains with preservation material	DNA/RNA Shield^TM^	6 × 1 mL aliquots	−80°C
						OMNI met®•GUT | ME-200	2 × 0.5 mL aliquots	
	Saliva	Baseline, week 1, 2, 3, 4, month 3, 6, 9, 12, 24	2 mL	Ambient	Sterile sample contains with preservation material	DNA/RNA Shield^TM^	2 × 1 mL aliquots	−80°C
	Bronchoalveolar lavage	Time of event requiring clinically directed BAL	40–80 mL	2–8°C, ice packs	Sterile sample container without preservation material	DNA/RNA Shield^TM^	20 × 1.5 mL aliquots	−80°C
	Colonoscopy	Time of event requiring clinically directed colonoscopy	40–80 mL	2–8°C, ice packs	Sterile sample container without preservation material	Stool—DNA/RNA Shield^TM^	10–20 × 1.5 mL aliquots	−80°C
			1–4 tissue samples	2–8°C, ice packs	Sterile sample container preservation material	Tissue—DNA/RNA Shield^TM^	1–4 × biopsy samples	
Healthy controls	Peripheral blood	Baseline	18 mL	2–8°C, ice packs	9 mL EDTA	Plasma	6 × 1.5 mL aliquots	−80°C
						PBMC	5 × 5 × 106/1 mL aliquots	Liquid nitrogen
	Stool	Baseline	25 g	Ambient	Sterile sample contains preservation material	DNA/RNA Shield^TM^	6 × 1 mL aliquots	−80°C
						OMNI met®•GUT | ME-200	2 × 0.5 mL aliquots	
	Saliva	Baseline	2 mL	Ambient	Sterile sample contains preservation material	DNA/RNA Shield^TM^	2 × 1 mL aliquots	−80°C
Deceased organ donors	Ascending and descending colon	Time of organ donation	10–20 cm of *en bloc* resected colon sample	2–8°C, ice packs	Sterile sample contains with preservation material	Stool—DNA/RNA Shield^TM^	6–12 × 1.5 mL aliquots	−80°C
						Stool - OMNI met®•GUT | ME-200	4 × 0.5 mL aliquots	
						Stool—Glycerol	4–6 2 mL aliquots	
						Tissue—DNA/RNA Shield^TM^	4 × 1–3 cm tissue aliquots	
	Bile	Time of organ donation	5–20 mL	2–8°C, ice packs	Sterile sample container without preservation material	DNA/RNA Shield^TM^	6 × 1.5 mL aliquots	−80°C
						OMNI met®•GUT | ME-200	2–4 × 0.5 mL aliquot	
						Glycerol	2–4 × 0.5 mL aliquot	

#### Laboratory data

Laboratory data will be obtained from the local biochemistry and haematology laboratory reporting systems at each participating site. Laboratory data will include full blood count, urea and electrolytes, liver function tests, C reactive protein and microbiological test results.

#### Baseline questionnaire

Prior to collection of samples, participants will be asked to complete a baseline questionnaire (box 1). This questionnaire has been adapted from an existing protocol for human gut microbiome sampling as part of the American Gut Project.^28^

Box 1 **HOMISPEC Participant Baseline Questionnaire**

**Personal information (please complete the following)**

1. What is your country of birth?

2. What is your race/ethnicity?

- Caucasian
- Aboriginal and Torres Strait Islander
- Asian
- Pacific Islander
- African
- Middle Eastern
- Other** Please explain in the supplemental answer section

Supplemental question regarding ethnicity

Race/ethnicity:

3. What is your weight (kg):

4. What is your height (cm)

5. How would you classify your diet?

- I eat anything with no exclusions (omnivore)
- I eat anything except red meat
- Vegetarian
- Vegetarian but eat seafood
- Vegan
- paleo-diet or primal diet
- modified paleo diet
- raw food diet
- FODMAP
- Westen-Price, or other low-grain, low processed food diet
- Kosher
- Halal
- Exclude nightshade vegetables (ie, white potatoes, tomatoes, eggplant, cayenne pepper, paprika, capsicums)
- Exclude dairy
- Exclude refined sugars
- Other restrictions not described here

6. How frequently do you take a probiotic? (any product specifically labelled as a ‘probiotic’. This does not include yoghurt or other dairy products)

- Never
- Rarely (a few times/month)
- Occasionally (1–2 times/week)
- Regularly (3–5 times/week)
- Daily

7. Do you smoke tobacco?

- Yes
- No

**General Information (please complete the following)**

8. I have travelled outside of Australia in the past……. (**If you answered a, b, or c, please explain in the supplemental answer section immediately after)

- Month**
- 3 months**
- 6 months**
- 1 year
- I have not been outside of Australia in the past year.

Supplemental question regarding Travel:

What countries:

How many days in each country:

9. How many people do you live with?

- None
- One
- Two
- Three
- More than three

10. Do you have pet(s) at home:

- Yes** Please explain in the supplemental answer section
- No

Supplemental answer regarding Pets:

Indoor/outdoor or confined:

Contact extent (ie, lick face, sleep in the bed with you etc):

11. On average, how many nights in a given week do you have an alcoholic beverage?

Answer:

12. How many alcoholic drinks do you usually have when you do drink?

- 1
- 1–2
- 2–3
- 3–4
- 4+
- I don't drink

13. How often do you brush your teeth?

- 2+ times/day
- 1–2 times/da
- Once a day
- Never
- I have dentures: either:
  - Single dentures
  - Top and bottom denture

14. How often do you floss your teeth?

- Daily
- Regularly (3–5 times/week)
- Occasionally (1–2 times/week)
- Rarely (few times/month)
- Never

**General Health Information (please complete the following)**

15. Have you ever had abdominal surgery?

- Yes
- No

If so, what surgery?

**Figure 2:**
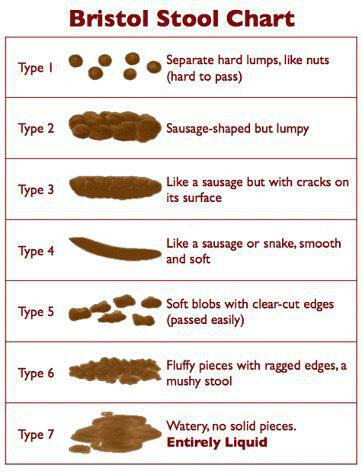
Bristol Stool Chart.

- Gallbladder removal
- Appendix removal
- Partial or total small bowel removal
- Partial or total large bowel removal
- Other:

16. How many times do you have a bowel movement in an average day?

- Less than one
- One
- Two
- Three
- Four
- Five or more

17. Describe the quality of your bowel movements^31^ (figure 2Figure 2):

- I tend to be constipated (types 1–2)
- I tend to have diarrhoea (watery stool) (types 6–7)
- I tend to have normal formed stool (types 3–5)

18. I have taken antibiotics in the last ______. (If you answered a or b, please indicate which antibiotic you took and what you were treating in the supplemental answer section)

- Week**
- Month**
- 6 months
- Year
- I have not taken antibiotics in the past year.

Supplemental answer regarding antibiotic/s:

Name:

Treatment for:

Duration:

19. My weight has _________ within the last 6 months.

- Increased more than 5 kg
- Decreased more than 5 kg
- Remained stable

#### Dietary data

Dietary intake will be self-reported by participants for the 48 hours prior to routine sample collection via the Keenoa mobile app. Keenoa is a smartphone image-based dietary assessment app that recognises and identifies food items using artificial intelligence and permits editing of food journal in real time.^29^

#### Clinical data

Baseline demographic information will be prospectively collected from the electronic medical record at each site. We will specifically gather information on the disease characteristics of each of the cohorts enrolled in addition ECOG Performance Status.^30^ Characteristics of hospitalisation during subsequent induction chemotherapy, allogeneic stem cell transplant, liver transplant or treatment with immune therapies will be collected.

#### Deceased organ donor samples and clinical data

Deceased organ donors will have deidentified tissue samples from the biliary and gastrointestinal tract made available through a research collaboration with the ADTB (HREC/48184/Austin-2019). Donor samples and basic demographic data routinely collected by the local organ procurement agency will be accessed (table 1).

### Study size

The HOMISPEC study aims to further our understanding of the host–microbe and the microbe–microbe interaction in the context of cancer treatment and transplantation. Due to the nature of a biorepository and an exploratory cohort study, not all research questions are predefined and will arise during the course of the study. The study will enrol over a 5-year period. An estimated 140 HSCT and 100 liver transplants are undertaken annually across the three sites. We estimate an 80% inclusion and 20% dropout rate across the different cohorts and anticipate up to 768 participants may be enrolled over the 5-year study period.

### Withdrawls

Participants will be free to withdraw from the study without consequence at any time and will be asked to sign a withdrawal of consent form indicating this. During the consent process, all participants will be informed that if they choose to withdraw after the analysis has been published, any deidentified data already uploaded to a publicly accessible database as part of a peer-review publication cannot be retracted. In these circumstances, the biological samples including any blood, stool, saliva or urine collected until the withdrawal will be analysed. Patients that withdraw from this cohort study will not be replaced. Any participants who have been enrolled but who do not meet all the prespecified time points for providing specimens and clinical data will be included in the analysis. This study is exploratory and hypothesis generating and therefore, while the sensitivity to detect outcomes will be reduced, the power of the study will be minimally affected.

### Data management

#### Data collection

Source data will be attributable, legible, contemporaneous, complete, consistent, original and accurate per the Australian Code for the Responsible Conduct of Research. Patient data will be reidentifiable at the site of recruitment with source data and then linked to a unique study number. A master list of participants will be retained by the Study Coordinator at each participating site. Only unique participant study numbers will be entered into the electronic case report forms (eCRFs). Patient data will be collected using eCRFs; these will be completed by qualified and authorised study personnel. The eCRFs will be hosted in a secure electronic data capture system (REDCap) that maintains a computer-generated, time-stamped audit trail hosted by the University of Melbourne. The electronic data collection system is password protected and only authorised study personnel will have access. Participant nutritional data collected via the Keenoa mobile app will be deidentified, encrypted and stored in a secure data capture system that maintains a computer-generated, time-stamped audit trail of all activity and for which there will be a formal research agreement. All samples are stored at −80°C or in liquid nitrogen and sample information is logged in a linked electronic database.

#### Data storage and preservation

Source data, which includes participant identifying information, will be kept within the swipe-card accessed Department of Infectious Diseases at Austin Health and Peter MacCallum Cancer Centre in a locked filing cabinet. Electronic CRFs will be securely stored for an indefinite period. Only authorised personnel have access to the database. This control extends to staff holding honorary appointments at the collaborating institution. The clinical research team at each site will be responsible for data collection under supervision of the site principal investigator. These data are stored on an encrypted server only accessible by the study investigators. Sequencing data will be stored in a deidentified format on a secure server and will only be accessible by the study investigators.

#### Data analyses

In the data analysis phase, only deidentified data pertinent to the study objectives will be used by named investigators at collaborating institutions. If there is a need for data analyses by an external collaborator, only deidentified data will be shared. Analysis will be conducted in accordance with the specified methods planned for this project ensuring the accuracy and validity of the results. Individual participants will not be identifiable from any presented or published material.

#### Data governance

The principal investigator will be the REDCap Data Custodian and will be responsible for the management of data generated and collected. As the custodian of the research data, the investigator is also accountable for the analysis and preservation of the data through to publication.

#### Data sharing

Study sites may choose to share deidentified patient data with external collaborators at other institutions following written consent from the lead investigator for those participants who consented to future research. A formal data-sharing agreement will be undertaken between the study site and any collaborators prior to transfer. Any future data-sharing agreement for future research will ensure data security and patient confidentiality is maintained.

#### Storage and access to the biorepository

All samples will be processed, deidentified and annotated with a unique laboratory code linked to clinical data and stored at −80°C in locked freezers or liquid nitrogen at either Austin Health or Peter MacCallum Cancer Centre (table 1). Access to laboratories and storage facilities is gained via swipe card access. Samples will be stored indefinitely for future research projects and accessed only by accredited staff involved in the processing and analysis of the samples. Patients will retain the right to have their samples removed from the biorepository and destroyed at any time by contacting the study doctor and return of the withdrawal of consent form. If the patient decides to have their samples destroyed, any data or analysis that were done before the request cannot be removed. However, no additional analysis will be done on remaining samples and all remaining samples will be destroyed. The principal investigator and the accredited research staff in the research laboratories at the Peter MacCallum Cancer centre and Austin Health are responsible for the destruction of the samples.

## Ethics and dissemination

The study has been approved by The Royal Melbourne Hospital Human Research Ethics Committee (HREC/61563/MH-2021) in Melbourne Australia with local site governance approval at Austin Health (SSA/61563/Austin-2021), Peter MacCallum Cancer Centre (SSA/61563/PMCC) and Melbourne Health (SSA/61563/MH-2021).

All eligible participants will be provided with a verbal explanation of the project and written information included in the consent form. One of the study investigators will thoroughly assess the participants competence and capacity to make an informed decision before the participants are recruited. All participants will be deemed competent if they (1) can comprehend and retain information relevant to making the decision, (2) understand the information and implications of the decision and (3) are able to evaluate the information and decide. For competent non-English speaking participants, an interpreter can be used as needed.

The final data set and biorepository will be the propriety of each recruiting site and a contractual agreement was signed between all participating sites and Melbourne Health. The investigational team will determine authorship concerning the International Committee of Medical Journal Editors guidelines. The results of this research project will be published and presented in various scientific forums without any identifying participant information. The data collected from the collaborating study sites mentioned will be analysed together and might serve for local practice change in the implicated hospitals. The protocol, deidentified participant-level data set and the statistical code will be available on request after the study is completed and findings published.

## References

[1] Registry ABMTR (2015). Australasian bone marrow transplant recipient registry: annual data summary 2015.

[2] Hernandez MDP, Martin P, Simkins J (2015). Infectious Complications After Liver Transplantation. Gastroenterol Hepatol (N Y).

[3] Schuster SJ, Bishop MR, Tam CS (2019). Tisagenlecleucel in Adult Relapsed or Refractory Diffuse Large B-Cell Lymphoma. N Engl J Med.

[4] Neelapu SS, Locke FL, Bartlett NL (2017). Axicabtagene Ciloleucel CAR T-Cell Therapy in Refractory Large B-Cell Lymphoma. N Engl J Med.

[5] Brudno JN, Kochenderfer JN (2016). Toxicities of chimeric antigen receptor T cells: recognition and management. *Blood*.

[6] Bupha-Intr O, Haeusler G, Chee L (2021). CAR-T cell therapy and infection: a review. Expert Rev Anti Infect Ther.

[7] Smith M, Dai A, Ghilardi G (2022). Gut microbiome correlates of response and toxicity following anti-CD19 CAR T cell therapy. Nat Med.

[8] Galloway‐Peña JR, Smith DP, Sahasrabhojane P (2016). The role of the gastrointestinal microbiome in infectious complications during induction chemotherapy for acute myeloid leukemia. Cancer.

[9] Peled JU, Gomes ALC, Devlin SM (2020). Microbiota as Predictor of Mortality in Allogeneic Hematopoietic-Cell Transplantation. N Engl J Med.

[10] Annavajhala MK, Gomez-Simmonds A, Macesic N (2019). Colonizing multidrug-resistant bacteria and the longitudinal evolution of the intestinal microbiome after liver transplantation. Nat Commun.

[11] Swarte JC, Li Y, Hu S (2022). Gut microbiome dysbiosis is associated with increased mortality after solid organ transplantation. Sci Transl Med.

[12] Liang D, Leung RK-K, Guan W (2018). Involvement of gut microbiome in human health and disease: brief overview, knowledge gaps and research opportunities. Gut Pathog.

[13] Rajagopala SV, Vashee S, Oldfield LM (2017). The Human Microbiome and Cancer. Cancer Prev Res (Phila).

[14] Taur Y, Xavier JB, Lipuma L (2012). Intestinal domination and the risk of bacteremia in patients undergoing allogeneic hematopoietic stem cell transplantation. *Clin Infect Dis*.

[15] Holler E, Butzhammer P, Schmid K (2014). Metagenomic analysis of the stool microbiome in patients receiving allogeneic stem cell transplantation: loss of diversity is associated with use of systemic antibiotics and more pronounced in gastrointestinal graft-versus-host disease. Biol Blood Marrow Transplant.

[16] Montassier E, Al-Ghalith GA, Ward T (2016). Pretreatment gut microbiome predicts chemotherapy-related bloodstream infection. Genome Med.

[17] Kato K, Nagao M, Miyamoto K (2017). Longitudinal Analysis of the Intestinal Microbiota in Liver Transplantation. Transplant Direct.

[18] Konuma T, Kohara C, Watanabe E (2020). Reconstitution of Circulating Mucosal-Associated Invariant T Cells after Allogeneic Hematopoietic Cell Transplantation: Its Association with the Riboflavin Synthetic Pathway of Gut Microbiota in Cord Blood Transplant Recipients. J Immunol.

[19] Kriss M, Verna EC, Rosen HR (2019). Functional Microbiomics in Liver Transplantation: Identifying Novel Targets for Improving Allograft Outcomes. Transplantation.

[20] 2017 NIH-wide microbiome workshop writing team (2019). 2017 NIH-wide workshop report on “The Human Microbiome: Emerging Themes at the Horizon of the 21st Century.”. Microbiome.

[21] Donnelly JP, Chen SC, Kauffman CA (2020). Revision and Update of the Consensus Definitions of Invasive Fungal Disease From the European Organization for Research and Treatment of Cancer and the Mycoses Study Group Education and Research Consortium. *Clin Infect Dis*.

[22] EBMT IDWPot (2001). Definitions of infectious diseases and complications after stem cell transplant.

[23] Teh BW, Mikulska M, Averbuch D (2024). Consensus position statement on advancing the standardised reporting of infection events in immunocompromised patients. Lancet Infect Dis.

[24] National Cancer Institute (U.S.) (2009). Common Terminology Criteria for Adverse Events (CTCAE). Rev. Ed.

[25] Harris AC, Young R, Devine S (2016). International, Multicenter Standardization of Acute Graft-versus-Host Disease Clinical Data Collection: A Report from the Mount Sinai Acute GVHD International Consortium. Biol Blood Marrow Transplant.

[26] (1997). Banff schema for grading liver allograft rejection: An international consensus document. Hepatology.

[27] Lee DW, Santomasso BD, Locke FL (2019). ASTCT Consensus Grading for Cytokine Release Syndrome and Neurologic Toxicity Associated with Immune Effector Cells. Biol Blood Marrow Transplant.

[28] McDonald D, Hyde E, Debelius JW (2018). American Gut: an Open Platform for Citizen Science Microbiome Research. mSystems.

[29] Ji Y, Plourde H, Bouzo V (2020). Validity and Usability of a Smartphone Image-Based Dietary Assessment App Compared to 3-Day Food Diaries in Assessing Dietary Intake Among Canadian Adults: Randomized Controlled Trial. JMIR Mhealth Uhealth.

[30] Oken MM, Creech RH, Tormey DC (1982). Toxicity and response criteria of the Eastern Cooperative Oncology Group. Am J Clin Oncol.

[31] Lewis SJ, Heaton KW (1997). Stool form scale as a useful guide to intestinal transit time. Scand J Gastroenterol.

