# Supplementary material for: Protocol for a clinically annotated biorepository of samples from Australian immune-compromised patients to investigate the host–microbiome interaction
Source: BMJ Open. 2024 Sep 12;14(9):e085504. doi: 10.1136/bmjopen-2024-085504 (PMC11440200; doi:10.1136/bmjopen-2024-085504)
Supplement: online supplemental table 1 [file bmjopen-14-9-s001.pdf]

Table 1. HOMISPEC participant timeline & evaluations

			**Day**				**Month**					**Other**
		**Baseline**	**0**	**7**	**14**	**28**	**3**	**6**	**9**	**12**	**24**	**Clinical events**
**Disease cohort Data**	Microbiome confounder questionnaire	X										
	Disease characteristics	X										
	Medication and antibiotic exposure	X	X	X	X	X	X	X	X	X	X	X
	Infections											X
	Immunological complications											X
	Survival										X	
	Dietary information	X	X	X	X	X	X	X	X	X	X	X
**Disease cohort Sampling**	Peripheral Blood	X	X	X	X	X	X	X	X	X	X	X
	Stool	X	X	X	X	X	X	X	X	X	X	X
	Saliva	X	X			X	X	X	X	X	X	X
	Colonoscopy/Bronchoalveolar lavage											X
**Deceased Organ Donors**	Demographics											
	Bile		X									
	Ascending and Descending colon		X									
**Healthy Controls**	Demographics		X									
	Dietary information		X									
	Peripheral Blood		X									
	Stool		X									
	Saliva		X									
