# Supplementary material for: Protocol for a clinically annotated biorepository of samples from Australian immune-compromised patients to investigate the host–microbiome interaction
Source: BMJ Open. 2024 Sep 12;14(9):e085504. doi: 10.1136/bmjopen-2024-085504 (PMC11440200; doi:10.1136/bmjopen-2024-085504)
Supplement: Supplementary file 2 [file bmjopen-2024-085504.reviewer_comments.pdf]

PEER REVIEW HISTORY

BMJ Open publishes all reviews undertaken for accepted manuscripts. Reviewers are asked to complete a checklist review form and are provided with free text boxes to elaborate on their assessment. These free text comments are reproduced below.

ARTICLE DETAILS

**TITLE (PROVISIONAL)**	PROTOCOL: PROTOCOL FOR A CLINICALLY ANNOTATED BIOREPOSITORY OF SAMPLES FROM AUSTRALIAN IMMUNECOMPROMISED PATIENTS TO INVESTIGATE THE HOST-MICROBIOME INTERACTION
**AUTHORS**	Smibert, Olivia; Trubiano, Jason; Kwong, Jason; Markey, Kate; Slavin, Monica

VERSION 1 - REVIEW

**REVIEWER NAME**	Koltun, Walter A.
**REVIEWER AFFILIATION**	Pennsylvania State University - Allentown Campus
**REVIEWER CONFLICT OF INTEREST**	None
**DATE REVIEW RETURNED**	30-Apr-2024

**GENERAL COMMENTS**	This is a proposed protocol for the development of biorepositories targeting the gut and bronchial microbiome, to be collected from three medical centers in Melbourne Australia. Patient groups will include patients with acute leukemia undergoing stem cell transplant, end stage liver disease/liver transplant and patients undergoing immunotherapy for cancer. It will also include specimens from deceased organ donors and healthy controls, as possible. Blood, stool and saliva samples are obtained at entry and then at regular intervals over 2 years, in addition to time points when significant clinic events occur. On the face of it, this will require a significant amount of work and logistics to coordinate amongst the many sites, for the multiple illnesses and for the collection and timely preservation of samples. The authors expect to recruit approximately 768 participants over 5 years.<br><br>Criticisms:<br>1) Though a specific clinical protocol is not expected of this publication format, it is not clear what the underlying or overall hypothesis is for this protocol. The authors suggest several possibilities, including that the gut(or lung) represents the SOURCE of microbes possibly causing deleterious clinical events, or possibly is it some immune compromise that occurs through a bacteria/host INTERACTION that alters patient homeostasis? Collecting blood and isolating PBMC is going to be done, but do these cells represent the site of such interaction? Is it not a greater probability that this takes place at the gut interface level by immune cells within the gut wall proper, which may not be represented in a peripheral sample? In addition, I assume the authors recognize that bacteria collected via fecal collection does not necessarily represent those found within the gut mucous layer, which are probably the more relevant microorganisms interacting with the host. I think if a more carefully defined but still overarching hypothesis was provided, it would allow a more careful and critical evaluation of the samples

	being collected and their validity for study. The dietary part of the questionnaire also suggests hypotheses that are separate from those above, but still not clearly rationalized, looking at the protocol.<br>2) It is not clear what the control populations will be even though they plan to recruit "healthy" patients. Clearly these healthy patients will not have bronchoscopies or colonoscopies for specimen collection, so how control specimens for those ill patients who do receive such scope procedures is unclear. Deceased donors will have actual tissue collected from the colon which will similarly allow for microbiome collection(including that from the mucous layer), but again, what will this be compared to? And how such specimens are collected in a reasonably fresh state so as to preserve the tissues and microbiome is not clear. Without a dedicated individual chaperoning this material, artifact from delay and inconsistent collection will be an issue. As I said, this is a logistical challenge, when so many patient groups and so many and variable collection points along the timeline occur, not to mention the several hospital sites, makes rigorous uniform collection of material difficult. The details regarding preservation and time constraints on patient self collection of specimens is also an issue. I might suggest a more narrowly defined patient group should be focused on, at least initially, to debug and streamline the process, to then expand to possibly other disease states. Especially since each of these various illnesses may have very different mechanisms and thus may require different sorts of biologic specimen collection<br>3) The clinical correlates to the specimen collections will be critical and goes well beyond the patient questionnaire provided. Again, the relevant clinical details will be different for each disease state and will probably only be refined as specific hypotheses are proposed. But the magnitude of this challenge should not be underestimated, especially since it probably can only be done by highly trained professionals, probably specialty MD's.<br>4) There will be unavoidably, significant missingness to the data collection, whether from the EMR or clinical specimens themselves. How will this be handled?<br>5) How will quality of specimen collection be measured and monitored? Cell viability? RIN?<br><br>I commend the authors on initiating the creation of such a biorepository, but the work effort will be immense and to avoid wasted effort, I would narrow the scope of the project overall, and have a unifying hypothesis in mind, that will better direct relevant specimen collection.

**REVIEWER NAME**	Leichtle, Alexander
**REVIEWER AFFILIATION**	University of Bern
**REVIEWER CONFLICT OF INTEREST**	n/a
**DATE REVIEW RETURNED**	28-May-2024

**GENERAL COMMENTS**	In their manuscript, the authors describe a study setup (HOMISPECT) to establish a biorepository of clinically annotated samples, which can be used to explore correlations between the gut microbiota and the immune system of immune-compromised patients.<br><br>General comments:

	1. The protocol addresses a growing field in medicine. Gut microbiota strongly depend on the hosts' immune system, and by collecting annotated samples, the authors might produce a valuable resource.<br>2. The multi centric nature of the study embraces the complexity of decentralized studies and is favorable over mono-centric designs.<br><br>Specifics:<br>1. Do the authors expect preanalytical differences between at-home-sampled samples and in-hospital-sampled ones?<br>2. Doesn't storage for 7 days at room temperature exert effects on the samples?<br>3. How are the lab values standardized (e.g. if centers use different tests)?<br>4. In terms of clinical EMR data I have concerns about the interoperability between centers. Is a standardized semantic (e.g. SNOMED CT) being used?<br>5. "Source data will be attributable, legible, contemporaneous, complete, consistent,<br>original and accurate" - How is this ensured?<br>6. Is de-identificiation sufficient to protect patients' privacy?<br>7. Who decides about the data/sample transfer from the study? Is there a governance structure set up?

VERSION 1 – AUTHOR RESPONSE

Reviewer Reports:

Reviewer: 1

Dr. Walter A. Koltun, Pennsylvania State University - Allentown Campus

Comments to the Author:

This is a proposed protocol for the development of biorepositories targeting the gut and bronchial microbiome, to be collected from three medical centers in Melbourne Australia. Patient groups will include patients with acute leukemia undergoing stem cell transplant, end stage liver disease/liver transplant and patients undergoing immunotherapy for cancer. It will also include specimens from deceased organ donors and healthy controls, as possible. Blood, stool and saliva samples are obtained at entry and then at regular intervals over 2 years, in addition to time points when significant clinic events occur. On the face of it, this will require a significant amount of work and logistics to coordinate amongst the many sites, for the multiple illnesses and for the collection and timely preservation of samples. The authors expect to recruit approximately 768 participants over 5 years.

Criticisms:

1) Though a specific clinical protocol is not expected of this publication format, it is not clear what the underlying or overall hypothesis is for this protocol. The authors suggest several possibilities, including that the gut(or lung) represents the SOURCE of microbes possibly causing deleterious clinical events, or possibly is it some immune compromise that occurs through a bacteria/host INTERACTION that alters patient homeostasis? Collecting blood and isolating PBMC is going to be

done, but do these cells represent the site of such interaction? Is it not a greater probability that this takes place at the gut interface level by immune cells within the gut wall proper, which may not be represented in a peripheral sample? In addition, I assume the authors recognize that bacteria collected via fecal collection does not necessarily represent those found within the gut mucous layer, which are probably the more relevant microorganisms interacting with the host. I think if a more carefully defined but still overarching hypothesis was provided, it would allow a more careful and critical evaluation of the samples being collected and their validity for study. The dietary part of the questionnaire also suggests hypotheses that are separate from those above, but still not clearly rationalized, looking at the protocol.

Thank you. The overall hypotheses of this study is that the interaction between the host and the microbiome is significant in the context of transplant and immune compromise. This is an early and evolving field of science and so this protocol is intended as a platform to undertake exploratory analyses of the interaction between the microbe and the host within this clinical context. The expected output from this work is therefore to summarise observations made of this interaction and then to generate further hypothesis that can be further explored in both subsequent observational cohorts (ie. More of less frequent sampling) or guide further preclinical exploration (i.e. exploring specific host-microbe interactions in a controlled setting in an animal models).

We agree that there are distinct differences between the luminal (stool) and mucosal associated gut microbiome (as there are differences in enteric microbiome at different stages along the length of the gut). Unfortunately, invasive mucosal sampling beyond clinically indicated colonoscopy samples is not feasible or appropriate in the clinical context of the patients with are enrolling. However, we expect to be able to contribute to the understanding of the differences between the stool luminal and mucosal associated microbiome in the context of transplant and immune compromise through our paired sample collection method which will include a stool and colonoscopy at the same clinical timepoint (stool collection prior to colonoscopy bowel preparation). The stool microbiome is a well-established and recognised sample for microbiome analyses as reported in publications in leading scientific journals including Nature, New England Journal of Medicine and Science, in spite of these known limitations, which we believe justifies this methodology.

The impact of diet as a confounder of microbiome observations in the setting of transplant and immune compromise is poorly understood and often not captured. The goal of collecting dietary information is to be able to adjust for this confounder during the microbiome analyses as robustly as possible. There are no specific hypotheses regarding diet and the microbiome as part of this study and no dietary interventions will be undertaken. This rationale has been added to the “Stengths and limitations” section

2) It is not clear what the control populations will be even though they plan to recruit "healthy" patients. Clearly these healthy patients will not have bronchoscopies or colonoscopies for specimen collection, so how control specimens for those ill patients who do receive such scope procedures is unclear. Deceased donors will have actual tissue collected from the colon which will similarly allow for microbiome collection(including that from the mucous layer), but again, what will this be compared to? And how such specimens are collected in a reasonably fresh state so as to preserve the tissues and microbiome is not clear. Without a dedicated individual chaperoning this material, artifact from delay and inconsistent collection will be an issue. As I said, this is a logistical challenge, when so

many patient groups and so many and variable collection points along the timeline occur, not to mention the several hospital sites, makes rigorous uniform collection of material difficult. The details regarding preservation and time constraints on patient self collection of specimens is also an issue.   I might suggest a more narrowly defined patient group should be focused on, at least initially, to debug and streamline the process, to then expand to possibly other disease states. Especially since each of these various illnesses may have very different mechanisms and thus may require different sorts of biologic specimen collection

Thank you. Further information regarding the selection of healthy controls is now provided in the participant eligibility criteria section.

Regarding additional samples collected from colonoscopy and bronchoscopy at time of infection and immune events. These samples cannot be paired with equivalent samples collected from healthy controls as we are not undertaking invasive sampling in the control cohort. However, as mentioned in the protocol, these samples will provide an opportunity to undertake exploratory analyses between samples collected from multiple compartments (stool, saliva and blood) to explore the association between both host and microbe during the time of infectious or immune events (i.e. stool, blood and bronchoscopy at time of pneumonia or stool, blood and colonoscopy at time of GVHD). The objective of collecting these additional event-driven samples is to support exploratory analyses to generate further hypotheses that may be interrogated in subsequent work. We are clear that this protocol is to establish the biorepository and is exploratory in its objectives.

Thank you for the comments regarding potential issues that may arise during organ donor sampling. We have provided further details regarding the procurement and transport of these samples to address the concerns raised. These tissues are collected by the transplant surgeon at the time of organ procurement and accompany the transplant organ with the surgeon back to the primary study site in static cold storage. This is a tightly controlled and time critical process and we do not anticipate significant variability between the time of samples procured and storage collected as part of this protocol. These samples will be processed and stored immediately on return to the transplant centre, minimising concerns regarding any potential artifacts introduced by delays.

3) The clinical correlates to the specimen collections will be critical and goes well beyond the patient questionnaire provided. Again, the relevant clinical details will be different for each disease state and will probably only be refined as specific hypotheses are proposed. But the magnitude of this challenge should not be underestimated, especially since it probably can only be done by highly trained professionals, probably specialty MD's.

Thank you. The definitions of clinical infection and immune compromise are now provided in the outcomes section. We also have highly trained specialty MDs as site PIs and sub investigators to ensure standardisation of data collection

4) There will be unavoidably, significant missingness to the data collection, whether from the EMR or clinical specimens themselves. How will this be handled?

We do not anticipate significant missing data. All patient cohorts continue follow up at the site of treatment/transplant as part of routine care indefinitely or until deceased.

We agree that there may be missingness of clinical samples. This is inevitable in a cohort of complex patients who may be unwell and unwilling to provide these voluntary stool samples. This is an inherent vulnerability in the study design and the authors will allow for this by recruiting a robust number of participants. We will minimise missingness by providing stool kits that are easy to use and train clinical ward staff to be able to collect samples for patients when they are admitted to hospital and may find stool collection challenging.

5) How will quality of specimen collection be measured and monitored? Cell viability? RIN?

Blood will be collected and immediately processed into plasma fractions and into PBMCs. Standard aliquots of both cell counts, and plasma fraction will be undertaken per the laboratory manual for the project which will be standardised across site. Stool samples will be batch processed weekly. Visual assessment of samples will be undertaken to ensure adequate sample volume prior to storage. DNA extraction will not be undertaken at time of storage but rather in batches at time of sequencing and analyses.

I commend the authors on initiating the creation of such a biorepository, but the work effort will be immense and to avoid wasted effort, I would narrow the scope of the project overall, and have a unifying hypothesis in mind, that will better direct relevant specimen collection.

Thank you. We are now already underway actively recruiting on this protocol. Fortunately, we have clinical and laboratory resourcing to support this work at each site. We believe the overall hypothesis is appropriate for this biorepository and anticipate that varied broad and more specific hypothesis will be able to be explored both across cohorts (i.e. common microbiome or host correlates of infection or immune event) or within cohorts (i.e. specific microbiota or host correlated of infection of rejection in liver transplant or allo SCT). The intention of this protocol is to be broad and allow the focus on different clinical cohorts over time as they emerge to answer clinically relevant questions – eg. Our initial focus is on liver transplant and CAR T while subsequent focus will be on other novel immune therapies.

Reviewer: 2

Dr. Alexander Leichtle, University of Bern

Comments to the Author:

In their manuscript, the authors describe a study setup (HOMISPECT) to establish a biorepository of clinically annotated samples, which can be used to explore correlations between the gut microbiota and the immune system of immune-compromised patients.

General comments:

1. The protocol addresses a growing field in medicine. Gut microbiota strongly depend on the hosts' immune system, and by collecting annotated samples, the authors might produce a valuable resource.

2. The multi centric nature of the study embraces the complexity of decentralized studies and is favorable over mono-centric designs.

Specifics:

1. Do the authors expect preanalytical differences between at-home-sampled samples and in-hospital-sampled ones?

Thank you. We do not expect major differences between stool samples collected in hospital or at home. Samples will be collected from both sites in a collection kit that contains a proprietary preservation liquid that facilitates storage at ambient temperature for 7 days without degradation of DNA per internal validation studies undertaken by the manufacturer and per the package insert. Samples will be collected either in hospital or express posted (overnight post) from patient collecting samples at home with the date of collected clearly time-stamped to allow batch processing within 7 days of collection.

2. Doesn't storage for 7 days at room temperature exert effects on the samples?

Thank you. As above

3. How are the lab values standardized (e.g. if centers use different tests)?

Thank you. The samples collection and processing kits and protocols we are utilising have been standardised across the two laboratories that will process samples for the three participating sites. Samples will not be processed in routine clinical labs but rather the research laboratories of the department at each participating site which allows the use of a consistent laboratory handbook for handling, processing and storage of samples.

4. In terms of clinical EMR data I have concerns about the interoperability between centers. Is a standardized semantic (e.g. SNOMED CT) being used?

Thank you. Clinical data is being collected manually from the EMR rather than being automatically extracted. It is correct that each site uses a separate EMR so manual extraction to ensure definitions are adhered to is essential. This information will be extracted by a clinician investigator. Internationally accepted definitions for infection and immune complications are provided in the protocol. All sites have a nominated data custodian who undertakes these responsibilities and reports to the site data governance committee.

5. "Source data will be attributable, legible, contemporaneous, complete, consistent,

original and accurate" - How is this ensured?

Thank you. Data will be stored and collected per the Australian Code for the Responsible Conduct of Research which articulate the broad principles and responsibilities that underpin responsible research conduct in Australia per the Australian Government National Health and Medical Research Counsel. Reference to this standard has now his has now been added to the manuscript.

6. Is de-identification sufficient to protect patients' privacy?

Per requirements of the Human Research Ethics Committee, it is acceptable for each site to host a reference list of patient identification data linked with a unique study number under password protection within the institutional firewall of each study site from which participants may be re-identified.

7. Who decides about the data/sample transfer from the study? Is there a governance structure set up?

Thank you. Further information has now been provided in the manuscript regarding the requirement for signed data sharing agreements to be executed prior to sharing patient data or samples with third-party collaborators. Governance oversight is provided by the Governance committee at the primary HREC site (Melbourne Health).

VERSION 2 – REVIEW

**REVIEWER NAME**	Leichtle, Alexander
**REVIEWER AFFILIATION**	University of Bern
**REVIEWER CONFLICT OF INTEREST**	n/a
**DATE REVIEW RETURNED**	30-Jul-2024

**GENERAL COMMENTS**	All comments/questions addressed adequately
